# Particle Ejection Contributions to the Rotational Acceleration and Orbit Evolution of Asteroid (101955) Bennu

**DOI:** 10.1029/2019JE006284

**Published:** 2020-03-11

**Authors:** D. J. Scheeres, J. W. McMahon, D. N. Brack, A. S. French, S. R. Chesley, D. Farnocchia, D. Vokrouhlický, R.‐L. Ballouz, J. P. Emery, B. Rozitis, M. C. Nolan, C. W. Hergenrother, D. S. Lauretta

**Affiliations:** ^1^ Smead Department of Aerospace Engineering Sciences University of Colorado Boulder Boulder CO USA; ^2^ Jet Propulsion Laboratory California Institute of Technology Pasadena CA USA; ^3^ Institute of Astronomy Charles University Prague Czech Republic; ^4^ Lunar and Planetary Laboratory University of Arizona Tucson AZ USA; ^5^ Department of Earth and Planetary Sciences University of Tennessee Knoxville TN USA; ^6^ Planetary and Space Sciences, School of Physical Sciences The Open University Milton Keynes UK

**Keywords:** YORP, Bennu, particle ejection, OSIRIS‐REx

## Abstract

This paper explores the implications of the observed Bennu particle ejection events for that asteroid's spin rate and orbit evolution, which could complicate interpretation of the Yarkovsky‐O'Keefe‐Radzievskii‐Paddack (YORP) and Yarkovsky effects on this body's spin rate and orbital evolution. Based on current estimates of particle ejection rates, we find that the overall contribution to Bennu's spin and orbital drift is small or negligible as compared to the Yarkovsky and YORP effects. However, if there is a large unseen component of smaller mass ejections or a strong directionality in the ejection events, it could constitute a significant contribution that could mask the overall YORP effect. This means that the YORP effect may be stronger than currently assumed. The analysis is generalized so that the particle ejection effect can be assessed for other bodies that may be subject to similar mass loss events. Further, our model can be modified to address different potential mechanisms of particle ejection, which are a topic of ongoing study.

## Key Points

- The net contribution of particle ejection from Bennu's surface on its spin rate and orbit evolution is minimal under some assumptions
- If there are systematics in ejection geometry, there may be meaningful contributions to rotational acceleration
- Our model can be tuned to address different potential mechanisms of particle ejection

## 1 Introduction

Among the many scientific discoveries made by the OSIRIS‐REx spacecraft's initial reconnaissance of asteroid (101955) Bennu, most were refinements and confirmations of expected results (Lauretta, DellaGiustina, et al., 2019). However, some may change our thinking about primitive asteroids such as Bennu. Prime among these was the observation of multiple particle ejection events from the Bennu surface, introduced in Hergenrother et al. (2019) and Lauretta, DellaGiustina, et al. (2019) and characterized in Lauretta, Hergenrother, et al. (2019). This discovery has launched a number of other investigations, this one included.

The effect of particle ejections can be classified as a nongravitational effect acting on the asteroid, which can provide a net transfer of angular and translational momentum to the asteroid. As both the Yarkovsky and the Yarkovsky‐O'Keefe‐Radzievskii‐Paddack (YORP) effects have been documented for this body (Chesley et al., 2014; Hergenrother et al., 2019; Nolan et al., 2019) and constitute scientific priorities for the OSIRIS‐REx mission, the net effect of the particle ejection events should be analyzed and placed into context.

Previous observations have indicated a steady increase in the rotation rate of Bennu that will lead to a doubling of its spin rate in about 1.5 million years (Hergenrother et al., 2019). This acceleration has been shown to be consistent with the YORP effect (Hergenrother et al., 2019; Nolan et al., 2019); however, to match the observed spin acceleration with physics‐based models required a combination of the Normal YORP effect, which predicts a deceleration of Bennu in general, and the Tangential YORP effect, which pushes the body to an acceleration mode (Nolan et al., 2019). The balance between these terms provides a small but positive acceleration to the body. Given the sensitivity of this observed spin acceleration to the different YORP effects, it is important to map out how particle ejections may affect this budget. It is possible to generate the measured rotational acceleration of Bennu by ejecting several particles of diameter ∼10 cm once per day in the westward direction from the equator (Lauretta, Hergenrother, et al., 2019), making it worthwhile to probe the possible effect of particle ejection on the asteroid's spin rate in more detail. Given the possible significance of particle ejections on spin state, we also briefly review whether these could have an influence on the translational motion of the asteroid, acting as a counterpart to the well‐documented Yarkovsky effect (Vokrouhlicky et al., 2015). We show that the effect of particle ejections should be negligible, consistent with the simple analysis reported in Lauretta, Hergenrother, et al. (2019); nonetheless, the details of this analysis merit discussion.

The particle ejection effect has similarities to the angular momentum drain and angular momentum splash effects analyzed in Dobrovolskis and Burns (1984) and Cellino et al. (1990), respectively. Those studies were focused on larger asteroids where impact ejecta speeds are close to surface escape speeds. Dobrovolskis finds that the effect of angular momentum drain is to decrease the spin rate of a satellite, with the rough proportion δΩ∼ΩδM/M, where δM is the overall mass loss from ejecta impacts, Ω is the spin rate of the asteroid, and δΩ is the change in spin rate. As δM<0, the overall spin rate decreases. Further analysis of this effect finds that it is most significant for asteroids of size ∼100 km, well outside of the Bennu scale (Pravec et al., 2002). This is driven by the usual ejecta speeds for solar system impacts, which in general exceed escape speed for smaller bodies. The angular momentum drain effect, in fact, becomes most effective when a large number of ejecta are given speeds that are on either side of the escape speed. We will comment on this phenomenon later in the paper, when we consider the possibility that the particle events are caused by impacting meteorites.

The spin rate of an asteroid such as Bennu can also be affected by the motion of surface boulders due to periods of rapid spin rate. This phenomenon has been recently studied in Brack and McMahon (2019) and is a similar mechanism by which mass movement affects the spin rate of an asteroid. As Bennu is not spinning rapidly enough to cause such surface motion, we do not consider this to be a contributor to the asteroid's spin rate evolution currently, although it may in the future should Bennu achieve a rapid spin state.

In this paper, we probe the influence of the particle ejection events on the spin rate of Bennu. First, we review the basic rotational dynamics model of Bennu and consider the influence of a detachable particle on its mass and rotation properties. We then consider the fate of ejected material and identify three distinct modes and the limits associated with them. Next, the influence of a single ejected particle is analyzed for each of these ejection modes to build a model of the effect of particle ejections. Then the overall influence of a distribution of such ejections is considered, both for a highly spatially focused set of ejections and for a model of uniform ejections. As part of this analysis, we find some analytical results that can be generalized to a wider class of asteroids that may be subject to a similar ejection phenomenon. The paper closes by discussing how the different hypothesized particle ejection models presented in Lauretta, Hergenrother, et al. (2019) can be modeled by the current approach.

## 2 Bennu and Particle Model Parameters

### 2.1 Bennu Parameters

The properties of Bennu needed for our study are given in Table 1, taking all values from Lauretta, DellaGiustina, et al. (2019). For its moments of inertia and gravity coefficients, Bennu is modeled as a constant density polyhedron following the approach outlined in Scheeres et al. (2019). The shape model provides the geometry of the surface (Barnouin et al., 2019) and in principle allows us to specify any point on the surface, r, and the local normal to the surface at that point, $\hat{n}$.

**Table 1:** Bennu Parameters (Lauretta, DellaGiustina, et al., 2019)

Parameter	Value	Units	Notes
GM	4.892	m ${}^{3}$/s ${}^{2}$	Gravitational parameter
M	$7.329\times 10^{10}$	kg	Total Bennu mass
ρ	1.190	g/cm ${}^{3}$	Bulk density
T	4.296057	h	Rotation period (J2000)
Ω	$4.062631\times 10^{-4}$	rad/s	Rotation rate (J2000)
$\dot{\Omega }$	$3.63\pm 0.52\times 10^{-6}$	degrees / day ${}^{2}$	YORP Acceleration
RA	85.65	deg	Right ascension (J2000)
DEC	−60.17	deg	Declination (J2000)
ϵ	177.6	deg	Computed obliquity
	Specific Principal Moments of Inertias		
$I_{xx}^{B}/M$	$2.2881\times 10^{4}$	m ${}^{2}$	Minimum Moment of Inertia
$I_{yy}^{B}/M$	$2.3649\times 10^{4}$	m ${}^{2}$	Intermediate Moment of Inertia
$I_{zz}^{B}/M$	$2.6794\times 10^{4}$	m ${}^{2}$	Maximum Moment of Inertia
$I^{B}/M$	$2.4\times 10^{4}$	m ${}^{2}$	Sphere‐equivalent Moment of Inertia
$C_{20}$	−0.05812		Oblateness coefficient
$C_{22}$	0.00320		Ellipticity coefficient
R	245.023	m	Normalizing and mean radius
$R_{min}$	230	m	Minimum averaged radius
$R_{max}$	270	m	Maximum averaged radius
$A_{s}$	$0.782\times 10^{6}$	m ${}^{2}$	Surface area
H	$8.0\times 10^{11}$	kg m ${}^{2}$/s	Total angular momentum

*Note.* Mass moments are computed from the published shape model (Barnouin et al., 2019) using a constant density assumption.

Of direct relevance for the current study is the measured rotational acceleration of Bennu's spin rate, which is consistent with the expected YORP effect. This was reported to be $3.63\pm 0.52\times 10^{-6}$ degrees/day ${}^{2}$ (Hergenrother et al., 2019). In natural units of radians and seconds, this becomes ${\dot{\Omega }}_{Y}∼8.49\pm 1.22\times 10^{-18}$ radians/s ${}^{2}$, leading to a YORP timescale of 1.5 Myr. Thus, even though measurable, it is an extremely small effect. Nonetheless, its effects over time make it important for interpreting the past and future evolution of this body. Thus, we must be careful properly to account for all orders of magnitude in our computations. A measure that we can use to determine the relevance of different effects is the fractional rate of change of the spin rate, or ${\dot{\Omega }}_{Y}/\Omega ∼2\times 10^{-14}$/s.

### 2.2 Particle Parameters

In addition to the Bennu model parameters, the model parameters for a generic particle must also be specified and are listed in Table 2. These parameters are chosen based on observations of the ejection events and the overall properties of the asteroid (Lauretta, DellaGiustina, et al., 2019; Lauretta, Hergenrother, et al., 2019). The particle's size is specified as a diameter d∈[0.5,5] cm, with a density ρ of 2 g/cm ${}^{3}$, based on Bennu meteorite analogs (1 et al., 2019), making it less porous than the overall asteroid (consistent with the estimated 50% porosity of the asteroid). The total mass of an individual grain is then $m=\pi \rho d^{3}/6\in [1.3\times 10^{-4},1.3\times 10^{-1}]$ kg. The overall moment of inertia of the particle, if modeled as a sphere, is $I^{P}=0.1\;m\;d^{2}=\pi \;\rho \;d^{5}/60\in [3.3\times 10^{-10},3.3\times 10^{-5}]$ kg m ${}^{2}$.

**Table 2:** Assumed Particle Parameters (Lauretta, Hergenrother, et al., 2019)

Parameter	Value	Units	Comments
ρ	2,000	kg/m ${}^{3}$	Grain density
d	0.5→5	cm	Particle diameter
m	$1.3\times 10^{-4}\to 1.3\times 10^{-1}$	kg	Particle mass range
$I^{P}$	$3.3\times 10^{-10}\to 3.3\times 10^{-5}$	kg m ${}^{2}$	Particle moment of inertia
v	$0\to 3^{+}$	m/s	Particle ejection speeds

Estimates based on observed events place the overall escaping mass loss rate and the rate for mass redistribution of ejecta that does not escape to have the same value of $10^{-7}$ kg/s, (Lauretta, Hergenrother, et al., 2019), with a relatively large uncertainty. Relating this to different‐sized particles, this is a loss rate or redistribution rate of about 66 0.5‐cm‐sized particles per day, eight 1‐cm‐sized particles per day, or one 5‐cm‐sized particle every 15 days.

A particularly effective way of modeling the mass redistribution and loss rate is to reformulate this for particle distributions. For a range of particle diameters d∈[0.5,5] cm, with a cumulative size frequency distribution where the cumulative number of particles greater than $d,N(>d)=d^{\alpha }$. For a reasonable range of α∈[−2,−4] and total number of ejected particles per year, $N\in [10^{3},10^{5}]$, we measure the total surface depth of the asteroid that is processed: This material either escapes or reimpacts the surface. The equivalent mass loss rates for combinations of α and N are shown in Table 3. These values bracket the nominal mass loss rate of $10^{-7}$ kg/s. Possible implications of this transfer rate on surface morphology and the changing spin rate are discussed in the final section.

**Table 3:** Equivalent Mass Loss Rate for Each Combination of Total Number of Particles Ejected, N, Modeled as a Power Law With d∈[0.5,5], and Cumulative Size Frequency Distribution, α

N	α	Mass loss rate (kg/s)
$10^{5}$	−2	$3.78\times 10^{-6}$
$10^{5}$	−3	$1.44\times 10^{-6}$
$10^{5}$	−4	$7.48\times 10^{-7}$
$10^{4}$	−2	$3.78\times 10^{-7}$
$10^{4}$	−3	$1.45\times 10^{-7}$
$10^{4}$	−4	$7.59\times 10^{-8}$
$10^{3}$	−2	$3.88\times 10^{-8}$
$10^{3}$	−3	$1.55\times 10^{-8}$
$10^{3}$	−4	$9.06\times 10^{-9}$

### 2.3 Comparisons Between Bennu and Particle Mass Properties

To place the relative size of a particle in context with that of the asteroid, comparisons can be made between their respective mass properties. Here we collect a number of them to justify assumptions that are made later in the paper. These revolve around the different mass and inertia properties of relevance to the analysis. The indicated ranges again trace back to the range of sizes for the particles.

(1) $$\begin{array}{ccc} \frac{m}{M} & \in [2\times 10^{-15},2\times 10^{-12}] & \\ \frac{mM}{m+M} & ∼m\left[1-\frac{m}{M}+\ldots \right]\\ \frac{I^{P}}{I_{zz}^{B}} & \in [2\times 10^{-25},2\times 10^{-20}]\\ \frac{mR_{max}^{2}}{I_{zz}^{B}-I_{xx}^{B}} & \in [3\times 10^{-14},3\times 10^{-11}]\\ \frac{mR_{max}^{2}}{I_{zz}^{B}} & \in [5\times 10^{-15},5\times 10^{-12}]\\ \frac{I^{P}}{mR_{max}^{2}} & \in [4\times 10^{-11},4\times 10^{-9}] \end{array}$$

## 3 Yarkovsky Effect

To compute the possible contribution of particle ejection to the Yarkovsky effect, we use a simple analysis. Given an ejected particle of mass m and speed v, the corresponding change in the asteroid's speed is ΔV∼−mv/M, where M is the asteroid's mass and ΔV is the change in Bennu's orbital velocity. Thus, for our given parameters, a particle ejected at 1 m/s will cause a change in the asteroid's speed of ∼ $1\times 10^{-11}$ m/s. Equating this to the computed Yarkovsky acceleration, this is equivalent to ejecting one 10‐cm particle per 5 s (in the correct direction), or about 18,000 particles per day.

Thanks to ground‐based optical and radar astrometry over two decades, the Yarkovsky effect has been detected with high statistical significance on Bennu (Chesley et al., 2014). In its simplest formulation, the Yarkovsky perturbation on Bennu is modeled as a transverse acceleration $A_{T}/r^{2.25}$, where $A_{T}=-9.2\times 10^{-13}$ m/s ${}^{2}$ and r is the heliocentric distance in AU, which ranges between 0.90 and 1.36 for Bennu. Thus, the magnitude of Yarkovsky acceleration ranges between $4.6\times 10^{-13}$ m/s ${}^{2}$ (at aphelion) and $1.2\times 10^{-12}$ m/s ${}^{2}$ (at perihelion), resulting in daily changes in velocity between $4\times 10^{-8}$ m/s and $1\times 10^{-7}$ m/s.

The linear momentum transfer from ejected particles is far smaller than that. Most detected particles are smaller than 10 cm in diameter with ejection velocities less than 1 m/s (Lauretta, Hergenrother, et al., 2019). Thus, as a worst case scenario, we consider a 10‐cm diameter particle with a density of 2 g/cm ${}^{3}$ escaping at 1 m/s in the transverse direction along the orbit of Bennu. The corresponding change in the velocity of Bennu would be $1.5\times 10^{-11}$ m/s, which is smaller by a factor of 3,000 to 7,000 than that caused daily by the Yarkovsky effect. Even though the contribution of particle ejections is far smaller than the Yarkovsky effect, we note that the Yarkovsky effect on Bennu is being detected with ever higher fractional precision (Chesley et al., 2003). The contribution of the linear momentum transfer from the ejected particles might become a consideration when modeling the Yarkovsky effect if this extreme level of precision is met.

Another aspect to be discussed is related to the dependence of the Yarkovsky effect on the rotation rate. We later discuss the effect of angular momentum transfer from ejected particles; there is the possibility that the change in rotation rate might alter the Yarkovsky effect. However, the full rotation acceleration detected (Hergenrother et al., 2019) would only cause a $7\times 10^{-5}$ fractional change in rotation rate of over 100 years, which is too small to affect current estimates of the Yarkovsky effect.

## 4 System Rotational Dynamics Properties

Now we note the main aspects of the rotational motion of the combined Bennu and particle system.

### 4.1 Combined Inertia Model

We first develop a combined mass model for Bennu and a particle. The particle is assumed to start at rest on the Bennu surface at a location r as measured from Bennu's center of mass. The asteroid is supposed to spin at a uniform rate Ω about its maximum moment of inertia. While we will eventually assume that m≪M, we cannot make this assumption too early lest we lose track of the effects that are of interest to us.

The center of mass of the combined bodies is assumed to be at the origin, giving us

(2) $$0=\frac{1}{M+m}\left[MR_{CM}+mr_{CM}\right].$$

Given the relative position vector between the bodies r, their relative centers of mass are $R_{CM}=-\frac{m}{M+m}r$ and $r_{CM}=\frac{M}{M+m}r$, the total mass being M+m.

The total inertia tensor of the system is $I=I^{B}+I^{P}-\mu \tilde{r}\cdot \tilde{r}$, where $\mu =\frac{Mm}{M+m},\tilde{r}$ is the cross‐product dyad associated with a vector $\text{r},\tilde{\text{r}}\cdot \tilde{\text{r}}=(\text{r}\text{r}-r^{2}U)$ and U is the identity dyad. Assume that the Bennu inertia tensor $I^{B}$ is in a principal axis coordinate frame, with the maximum moment of inertia about the $\hat{z}$ axis. As the particle is modeled as a sphere, its inertia tensor is $I^{P}U$. For definiteness, assume that the particle has a location on the $\hat{x}$‐ $\hat{z}$ plane at a radius r and latitude l. Then the total inertia tensor in the Bennu principal axis frame is

(3) $$I=\left[\begin{array}{ccc} I_{xx}^{B}+I^{P}+\mu r^{2}\sin^{2}l & 0 & -\mu r^{2}\cos l\sin l\\ 0 & I_{yy}^{B}+I^{P}+\mu r^{2} & 0\\ -\mu r^{2}\cos l\sin l & 0 & I_{zz}^{B}+I^{P}+\mu r^{2}\cos^{2}l \end{array}\right].$$

For the combined system, the maximum moment of inertia axis is rotated about the $\hat{y}$ axis by an angle θ, given by the equation

(4) $$\tan2\theta =-\frac{\sin2l\mu r^{2}}{I_{zz}^{B}-I_{xx}^{B}+\cos2l\mu r^{2}}.$$

The principal moments of inertia are computed to be

(5) $$\begin{array}{ccc} I_{xx} & =\frac{1}{2}\left[I_{zz}^{B}+I_{xx}^{B}+2I^{P}+\mu r^{2}\right]-\frac{1}{2}\left[I_{zz}^{B}-I_{xx}^{B}+\mu r^{2}\right] & \\ & \;\times \sqrt{1-4\sin^{2}l\frac{\frac{\mu r^{2}}{I_{zz}^{B}-I_{xx}^{B}}}{{\left(1+\frac{\mu r^{2}}{I_{zz}^{B}-I_{xx}^{B}}\right)}^{2}}}, \end{array}$$

(6) $$I_{yy}=I_{yy}^{B}+I^{P}+\mu r^{2},$$

(7) $$\begin{array}{ccc} I_{zz} & =\frac{1}{2}\left[I_{zz}^{B}+I_{xx}^{B}+2I^{P}+\mu r^{2}\right]+\frac{1}{2}\left[I_{zz}^{B}-I_{xx}^{B}+\mu r^{2}\right] & \\ & \;\times \sqrt{1-4\sin^{2}l\frac{\frac{\mu r^{2}}{I_{zz}^{B}-I_{xx}^{B}}}{{\left(1+\frac{\mu r^{2}}{I_{zz}^{B}-I_{xx}^{B}}\right)}^{2}}}. \end{array}$$

In the above expressions, $\frac{\mu r^{2}}{I_{zz}^{B}-I_{xx}^{B}}∼\frac{m\;r^{2}}{I_{zz}^{B}-I_{xx}^{B}}\ll 1$, and thus, we can expand the angle and the moment of inertia formula to first order to get the estimated correction. The angle condition (4) simplifies to

(8) $$\tan2\theta ∼-\frac{m\;r^{2}}{I_{zz}^{B}-I_{xx}^{B}}\sin2l$$

while the $I_{xx}$ and $I_{zz}$ moments of inertia become

(9) $$I_{xx}∼I_{xx}^{B}+I^{P}+m\;r^{2}\sin^{2}l,$$

(10) $$I_{zz}∼I_{zz}^{B}+I^{P}+m\;r^{2}\cos^{2}l.$$

Finally, $I^{P}\ll m\;r^{2}$ and thus can be neglected in these expressions. This yields the final approximations to the principal moments of inertia for the combined system

(11) $$I_{xx}∼I_{xx}^{B}+mr^{2}\sin^{2}l,$$

(12) $$I_{yy}∼I_{yy}^{B}+mr^{2},$$

(13) $$I_{zz}∼I_{zz}^{B}+mr^{2}\cos^{2}l.$$

In a tensor format the approximate total inertia of the system is then specified as

(14) $$I=I^{B}-m\tilde{r}\cdot \tilde{r}.$$

### 4.2 Total Angular Momentum and the Effect of Changes

Now suppose that the Bennu and particle system rotates at a uniform rate Ω about its maximum moment of inertia. Then the total angular momentum is H=I·Ω. As the rotation is about a principal axis, the magnitude of the angular momentum is just $H=I_{zz}\Omega$, using the corrected $I_{zz}$ from above, to get $H=\left[I_{zz}^{B}+mr^{2}\cos^{2}l\right]\Omega$. The angle between the principal axis and the Bennu ${\hat{z}}^{B}$ axis is the angle θ, whose magnitude was bounded above. We can use this bound to constrain the dot product between the principal axis of inertia and the Bennu principal axis, $1-\hat{z}\cdot {\hat{z}}^{B}\ll 1\times 10^{-20}$.

A key aspect of our study is the effect of a small deviation in the total angular momentum from this equilibrium state. When subjected to a small change in angular momentum, defined as ΔH, the system enters a complex rotation state in general. The body's complex rotation state can be captured with two numbers, its effective rotation rate $\Omega_{l}$ and its dynamic inertia $I_{D}$. These are defined as the equivalent spin rate and moment of inertia of a sphere with the total kinetic and angular momentum of the given spin state. Or, given the system kinetic energy T and angular momentum H, these parameters are defined as $T=I_{D}\Omega_{l}^{2}/2$ and $H=I_{D}\Omega_{l}$, or $\Omega_{l}=2T/H$ and $I_{D}=H^{2}/(2T)$.

These quantities define the complex rotation state, and the details of the body's nutation and precession can be directly solved from these quantities. As such, they become appropriate ways to track the current rotation state of the system. In terms of a small deviation of angular momentum from uniform rotation about the maximum moment of inertia, these are computed as (Scheeres et al., 2000)

(15) $$\Omega_{l}∼\Omega +\frac{\Delta H_{z}}{I_{zz}}+\frac{1}{2I_{zz}^{2}\Omega }\left[\frac{2I_{zz}-I_{xx}}{I_{xx}}\Delta H_{x}^{2}+\frac{2I_{zz}-I_{yy}}{I_{yy}}\Delta H_{y}^{2}\right]+\ldots ,$$

(16) $$I_{D}∼I_{zz}-\frac{1}{I_{zz}\Omega^{2}}\left[\frac{I_{zz}-I_{xx}}{I_{xx}}\Delta H_{x}^{2}+\frac{I_{zz}-I_{yy}}{I_{yy}}\Delta H_{y}^{2}\right]+\ldots .$$

If we assume that the asteroid dissipates this complex motion and settles back into uniform rotation, then $I_{D}=I_{zz}$ with the second‐order term in equation (16) going to zero, and the overall change in the total rotation rate relaxes to

(17) $$\Delta \Omega =\frac{\Delta H_{z}}{I_{zz}}+\frac{1}{2I_{zz}^{2}\Omega }\left[\Delta H_{x}^{2}+\Delta H_{y}^{2}\right]+\ldots .$$

This shows that even if the average effect of ejections yields a net zero change in the z‐axis angular momentum, the effect of adding angular momentum transverse to the maximum moment of inertia will tend to spin up the body. This formula assumes that the system relaxes to uniform spin between every ejection. If this is not the case, then the total net angular momentum should be averaged first, then the net change in terms should be applied.

## 5 The Fate of Particle Ejecta and Implications

Based on observations of Bennu's ejected particles (Lauretta, Hergenrother, et al., 2019), we have defined the general range of sizes that interests us. Their ejection speeds range from near 0 to above 3 m/s. Across this range of speeds, we find very different final outcomes, which are studied in detail in McMahon et al. (2019). These can be classified as immediate reimpact, eventual reimpact, eventual escape, or immediate escape. Each of these outcomes has a different implication for how the Bennu spin state may be changed.

Our general model for a particle ejection provides a location on the asteroid surface r, a launch speed v, a launch direction $\hat{v}$, and a particle mass m. The launch velocity will be defined in terms of the local surface normal $\hat{n}$ and a local cone and clock angle δ and λ, such that $\hat{v}=\cos\delta \hat{n}+\sin\delta \cos\lambda {\hat{t}}_{1}+\sin\delta \sin\lambda {\hat{t}}_{2}$, where ${\hat{t}}_{1}=\hat{z}\times \hat{n}/|\hat{z}\times \hat{n}|$ is the unit vector pointing to the local East on the surface of the asteroid and ${\hat{t}}_{2}=\hat{n}\times {\hat{t}}_{1}$ is the unit vector pointing locally to the North (we note that these must be redefined at the poles and that these do not correspond to true East or North). This provides a general way to specify the ejection location and direction using the existing shape model of Bennu. Once a particle of mass m is lofted, it affects the total translational and rotational momentum of the asteroid. For the current study, we only focus on the relative change in the asteroid's momentum and thus the relative system of Bennu and a mass particle.

### 5.1 Separation Angular Momentum Budget

Consider our system of total angular momentum with Bennu and a particle on its surface:

(18) $$\text{H}={\text{I}}_{B}\cdot \Omega -m\tilde{r}\cdot \tilde{r}\cdot \Omega ,$$

where, as discussed above, we ignore the moment of inertia of the particle and the higher‐order contributions to the system moment of inertia. We suppose that the particle is ejected from the surface with a velocity v while the total angular momentum of the system stays constant. In our budget, Bennu then spins with a new angular velocity, $\Omega^{\prime }$, and the particle's total angular momentum adds to its initial value. Thus, we obtain the balance equation

(19) $${\text{I}}_{B}\cdot \Omega -m\tilde{r}\cdot \tilde{r}\cdot \Omega ={\text{I}}_{B}\cdot \Omega^{\prime }-m\tilde{r}\cdot \tilde{r}\cdot \Omega +mr\times v.$$

Canceling terms, we find the instantaneous change in the Bennu angular momentum to be

(20) $$\Delta H^{B}=-mr\times v.$$

This is the key relationship in our system and can be used to track the overall change in rotation state of the system. In general, the new angular velocity is misaligned with the principal axis, and thus, the body has a very small tumbling motion (with a nutation angle on the order of θ as bounded earlier). If dissipation is not present, the body will continue its slightly perturbed complex rotation until the next particle ejection, at which time the system will suffer another impulsive update to its angular momentum.

### 5.2 Reimpacting Ejecta

The above relations assume that there is no further interaction between the ejected particle and Bennu; however, this is often not the case. The simplest situation is that the particle reimpacts the asteroid. If the particle has been given no additional angular momentum due to exogenous effects, then the total angular momentum of the system is conserved, although energy is lost. In a closed system, momentum is conserved, although energy may not be and generally decreases. Specifically, the ejection of the particle can be due to mechanical energy being released; however, if the particle eventually settles back on the surface and is stationary, then the total mechanical energy of the system has been decreased. If a particle is ejected and eventually settles on the surface at a different location, then the main change in the system is a change in the inertia of the asteroid and particle system and an induced change in spin rate and spin state. If the initial angular momentum is

(21) $$\text{H}=\left[{\text{I}}_{B}-m\tilde{r}\cdot \tilde{r}\right]\cdot \Omega ,$$

then the final angular momentum is equal, but with a different location for the particle and a different spin rate

(22) $$\text{H}=\left[{\text{I}}_{B}-m\tilde{r^{\prime }}\cdot \tilde{r^{\prime }}\right]\cdot \Omega^{\prime }.$$

More simply, we obtain $I\cdot \Omega =I^{\prime }\cdot \Omega^{\prime }$. If we assume that the changes in the inertia matrix and the spin rate are both small, then we can ignore higher‐order contributions, and we find the simplified result

(23) ΔI·Ω=−I·ΔΩ.

As the relative change in moment of inertia is small, the net change in spin rate is also small. Also, as the body relaxes back to principal axis rotation, the only systematic change is due to the change in $I_{zz}$. In the case of mass movement, this could potentially either increase or decrease. As it is expected that particles preferably reimpact and are trapped in the equatorial region (McMahon et al., 2019), this effect should tend to increase $I_{zz}$ and hence decrease the spin rate assuming a fixed angular momentum. The overall change in $I_{zz}$ due to a particle migration is

(24) $$\Delta I_{zz}=-m\hat{z}\cdot \left[\tilde{r^{\prime }}\cdot \tilde{r^{\prime }}-\tilde{r}\cdot \tilde{r}\right]\cdot \hat{z}$$

(25) $$=m\left[r^{\prime 2}-r^{2}-{\left(r\prime \cdot \hat{z}\right)}^{2}+{\left(r\cdot \hat{z}\right)}^{2}\right]$$

(26) $$=m\left[x^{\prime 2}+y^{\prime 2}-x^{2}-y^{2}\right]$$

(27) $$=m\left[r_{H}^{\prime 2}-r_{H}^{2}\right],$$

where $r_{H}$ is the horizontal radius, as measured from the spin axis. If the trend is for $r_{H}^{\prime }>r_{H}$, meaning that the particles tend to move away from the spin axis and toward the equator, then the systematic trend is for the moment of inertia to increase, causing the overall spin rate to decrease.

This provides an estimate for the change in spin rate due to a reimpacting particle. Assuming that the system has relaxed back to uniform spin again, this is

(28) $$\Delta \Omega ∼-\frac{m\left(r_{H}^{\prime 2}-r_{H}^{2}\right)}{I_{zz}^{B}}\Omega .$$

We can bound this by assuming that the particle was ejected at the pole and landed at the equator, where we have the maximum radius. Then the fractional change in spin rate ranges from $-5\times 10^{-15}$ to $-5\times 10^{-12}$, from equation (1). The fractional rate of change of the Bennu spin rate is on the order of $2\times 10^{-14}$/s, and thus, if the migration of particles occurs frequently, this effect may be meaningful. To quantify, we can divide $\left(|\Delta \Omega /\Omega |)/({\dot{\Omega }}_{Y}/\Omega \right)$ to find the rate of particle resettling to produce the same magnitude as the measured YORP effect. Corresponding to the size range considered, this equality occurs if particles migrate every 0.25 to 250 s, orders of magnitude larger than the reported mass movement rate.

### 5.3 Temporary Orbit

If a particle does not immediately escape or reimpact, then in general its orbit must be changed due to exogenous forces or by interactions with the spinning Bennu gravity field. We treat these two cases separately.

#### 5.3.0.1 Exogenous Forces

If the particle experiences an exogenous force that changes its orbit but does not lead to further interactions with Bennu, then the basic equation for the Bennu angular momentum change, equation (20), still holds. The total orbital angular momentum of the particle when it enters orbit is

(29) $$H_{P}=mr\times \left[\Omega \times r+v\right]$$

(30) $$=mr\times v_{I}.$$

Owing to interactions with exogenous forces, this angular momentum can change over time. If the body ultimately escapes or remains in orbit without impacting, owing solely to exogenous effects, then the change in Bennu angular momentum and spin rate are just due to the initial ejection. If the particle ultimately reimpacts the surface, then the impact angular momentum will, in general, be different than the ejected angular momentum, yielding, in the body space,

(31) $$H_{P}^{\prime }=mr^{\prime }\times \left[\Omega^{\prime }\times r^{\prime }+v^{\prime }\right],$$

where we take the “new” body spin rate. The position is changed but is still on the body. The reimpact speed with respect to the body is also changed. In this situation, there is a net transfer of angular momentum to the particle‐Bennu system, giving a balance between the initial and the final angular momentum. On impact, the new system angular momentum is

(32) $$H^{\prime }=\left[{\text{I}}_{B}-m{\tilde{r}}^{\prime }\cdot {\tilde{r}}^{\prime }\right]\cdot \Omega^{\prime }+mr^{\prime }\times v^{\prime }.$$

At this point, we can compare it to the angular momentum after the particle ejection, under the assumption that the Bennu angular momentum has not been changed in the meantime. Thus,

(33) $$H=\left[{\text{I}}_{B}-m\tilde{r}\cdot \tilde{r}\right]\cdot \Omega^{\prime }+mr\times v.$$

Then, subtracting these two to obtain the total change in angular momentum yields

(34) $$\Delta H∼H_{P}^{\prime }-H_{P},$$

which adds the angular momentum change experienced by the particle due to exogenous forces to the total system. Thus, the overall system experiences a change in its angular momentum. Also, the moment of inertia may be changed as a result of redistribution of material. Then, given the new landing location and the incoming speed, the change in angular momentum, and ultimately the change in spin, can be computed.

The maximum change in angular momentum can be estimated. Let us assume that the particle initially ejects with its inertial velocity aligned with its radius, giving a zero angular momentum inertially, and then at reimpact is on a parabolic orbit with periapsis at the surface of the asteroid. Then the total change in angular momentum can be estimated as $\Delta H_{P}∼m\sqrt{2GMR}$. For Bennu, this takes on a value of $\Delta H_{P}∼6.3\times 10^{-3}\to 6.3$ kg m ${}^{2}$/s. In terms of the total angular momentum of Bennu, this represents a small fraction, $8\times 10^{-15}\to 5\times 10^{-12}$.

If we focus on the final spin rate of the asteroid, after relaxation, then we can use our equations to solve for the total change in spin rate as

(35) $$\Delta \Omega =-\frac{m\left(r_{H}^{\prime 2}-r_{H}^{2}\right)}{I_{zz}^{B}}\Omega +\frac{\Delta H_{z}^{P}}{I_{zz}^{B}}+\ldots .$$

Here we see that the effect is similar to immediate reimpact, with the added change in angular momentum. There are no immediately clear rules on whether there is a systematic change in the orbit angular momentum.

#### 5.3.0.2 Internal Effects

If the ejected particle remains in orbit about the asteroid, this can also be due to an exchange of angular momentum between the two bodies, as the particle interacts with the rotating gravity field of the asteroid. Such interactions can be important: If the particle is eventually ejected it will cloud the net contribution of the ejected particle to the eventual change in spin rate of the asteroid.

We can estimate and bound the amount of angular momentum transferred to the asteroid by such interactions. First, we only wish to track changes in the z axis of angular momentum, as these will directly affect the spin rate at first order. This means that the $J_{2}$ coefficient does not contribute and only the $C_{22}$ coefficient does, which is already relatively small for Bennu. An ideal evaluation is made in Scheeres et al. (2000), which models the change in angular momentum of an asteroid during a disruption. Using equation (22) from that text and assuming the ν≪1,δ=0, and $V_{\infty }=0$ yields

(36) $$\Delta H_{z}∼2\sqrt{2}\sqrt{\frac{GM}{R^{3}}}C_{22}R^{2}m\sin(2\lambda )$$

and hence the change in spin rate

(37) $$\Delta \Omega ∼5\sqrt{2}\sqrt{\frac{GM}{R^{3}}}C_{22}\frac{m}{M}\sin(2\lambda ),$$

where $C_{22}$ is the unnormalized coefficient and λ is the longitude along which the particle is ejected. For Bennu, the magnitude of this effect can be estimated to be

(38) $$\Delta \Omega ∼\left(1.6\times 10^{-15}\to 1.6\times 10^{-12}\right)\sin(2\lambda ).$$

This fluctuation was computed for an ideal case where the particle moves away from the body, and as the particle approaches, that change in angular momentum will reverse in sign. Assuming that these events are randomly distributed in longitude, this shows us that not only is the magnitude vanishingly small but it should in general be averaged out over time. Once a particle has escaped from the system after an extended period of interaction, the angular momentum that it takes away with it is no longer directly related to its initial angular momentum but is changed by these sequences of interactions. The interactions with $J_{2}$ also change the angular momentum, but only about the axes perpendicular to the spin axis, which have a smaller effect on the final spin rate.

### 5.4 Direct Escape

Finally, we evaluate the effect of a direct escape on the system. This occurs if the particle is launched with sufficient speed so that it is on a hyperbolic orbit and will escape from the system, carrying its angular momentum away with it. Then the net change in Bennu angular momentum is −mr×v. Of most interest now is to solve for the necessary speed relative to the asteroid surface that will ensure that the particle escapes.

**Figure 1:**
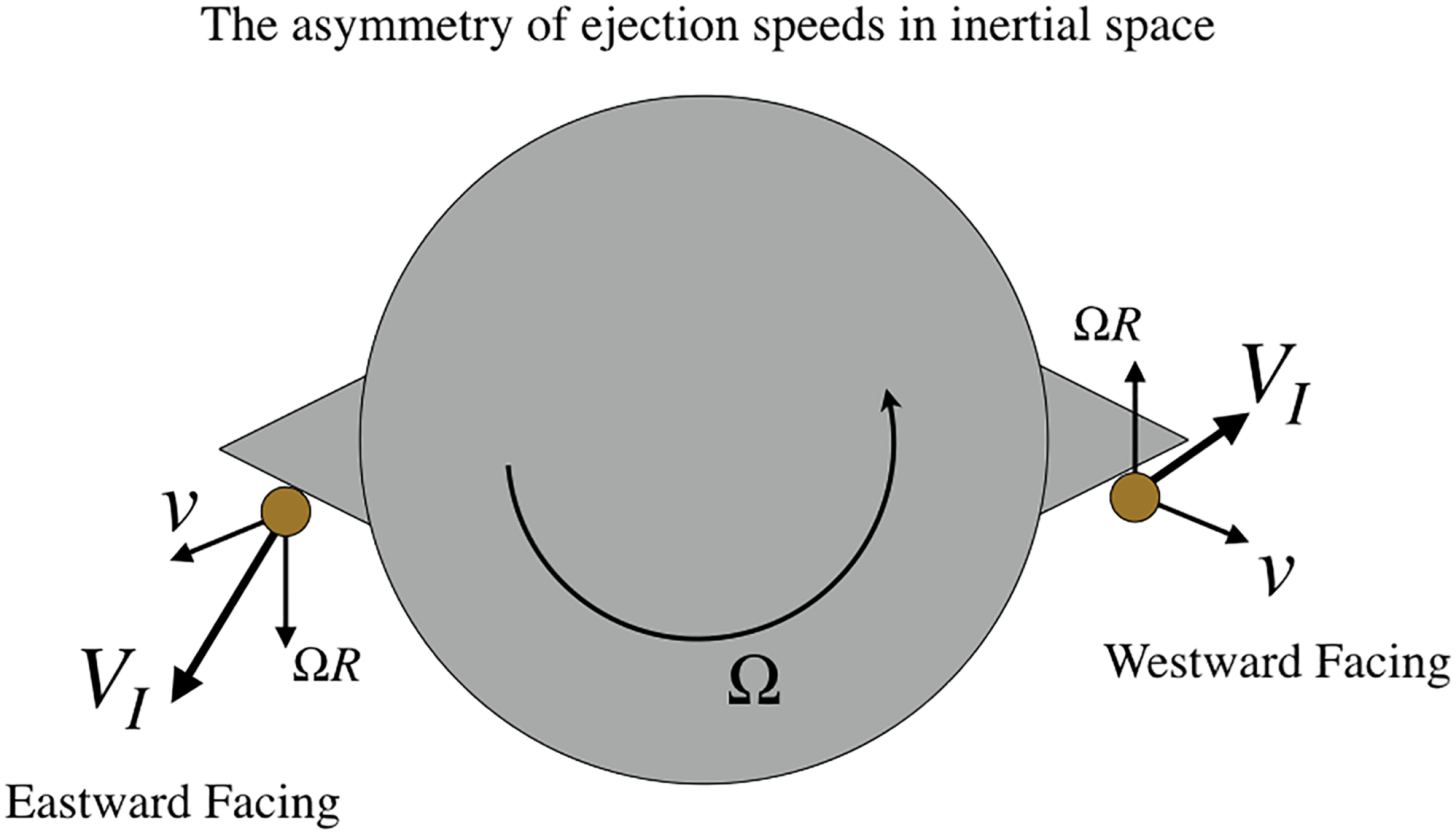
Inertial speeds after ejection are a strong function of location on a rotating body, making it possible for eastward facing particles to escape at a lower ejection speed than westward facing particles.

First consider the inertial speed of a particle resting on the asteroid surface, which is $v_{I}=\Omega \times r$ (Figure 1). Depending on the surface topography in relation to the spin state, this velocity can point away from the asteroid surface if the point is on the leading edge of the asteroid or may point into the asteroid if it is on a trailing edge. Because of this, the necessary additional speed necessary to impart escape speed to a particle may vary substantially over a small body's surface.

The escape speed from a point on the asteroid surface is then defined as the additional speed normal to the asteroid surface that would provide the particle sufficient speed to have a zero Keplerian energy when far from the asteroid. This criterion has been tested in earlier papers (Scheeres et al., 1996, 1998) and found to be a reliable indicator, sufficient to develop surface maps to follow trends across an asteroid's surface. This definition results in a velocity vector in inertial space of $v_{I}=v_{esc}\hat{n}+\Omega \times r$. Our definition requires the magnitude of $v_{I}$ to be greater than or equal to $\sqrt{2U_{max}}$ where $U_{max}=\max\left[U(r),GM/r\right]$, where U(r) is the gravitational force potential of the asteroid, which is a conservative result. Solving for the surface escape speed $v_{esc}$ yields (Scheeres et al., 1998)

(39) $$\begin{array}{ccc} v_{esc}(r) & =-\hat{n}\cdot (\Omega \times r) & \\ & \;+\sqrt{{\left[\hat{n}\cdot (\Omega \times r)\right]}^{2}+2U_{max}(r)-{\left(\Omega \times r\right)}^{2}}. \end{array}$$

**Figure 2:**
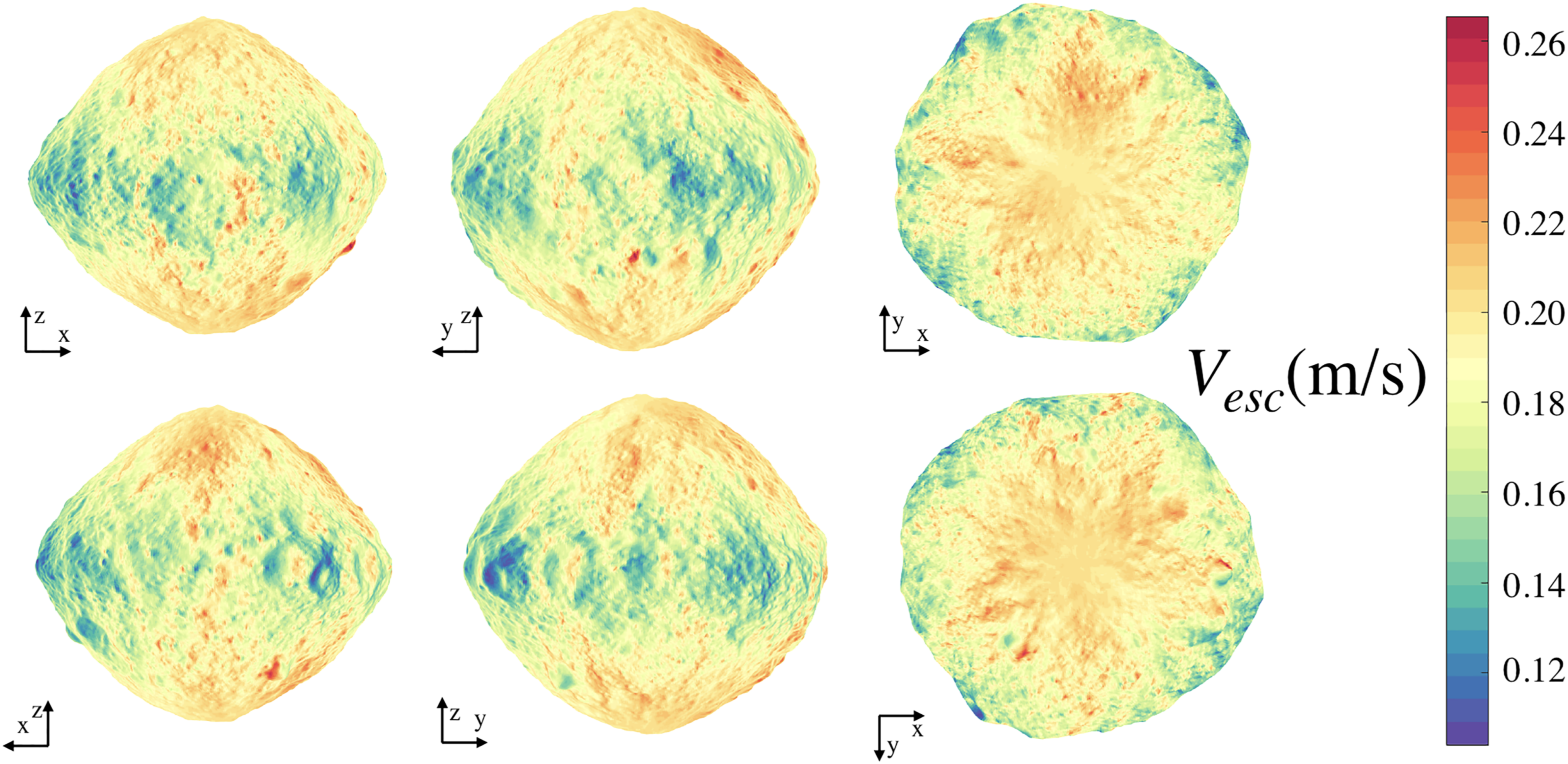
Escape speed mapped over the surface Bennu.

This is not a well‐defined quantity when the body has locally nonconvex regions where a speed normal to the surface would result in a reimpact with a different region of the body. Despite this, the result still indicates the level of speed necessary to generate energies consistent with escape from a body. These speeds have been computed for Bennu and are shown in Figure 2, where we see that they vary from ∼10 to 27 cm/s (Scheeres et al., 2019).

## 6 Integrated Effects of Particle Ejections

Given each model outlined above, we can evaluate the net effect of many particle ejections, including considerations of systematic directionality. For each of the effects considered, the effect of the particle ejection can be evaluated either as a change in overall relaxed spin rate or as a change in Bennu rotational angular momentum followed by relaxation. The most important secular contribution for the asteroid spin rate is along the $\hat{z}$ axis of the body, with the changes in angular momentum along the transverse axes yielding a much smaller effect. Thus, in the following, we only focus on $\Delta H_{z}=\hat{z}\cdot \Delta H$. We will discuss each case in detail but first establish some results.

### 6.1 Random Accumulation

Several of the ejection modes allow for the accumulation of a random component of angular momentum change. There are two ways for this to occur. First, a sequence of particle ejections may occur in rapid succession, with the system being unable to relax to uniform rotation. This leads to the net angular momentum being summed over multiple events, and then having the system relax to its minimum energy state. Conversely, the system may relax after each particle ejection, which would then require the net change to the final spin rate to be computed. We will discuss both extremes below, realizing that the actual situation may lie between these extremes.

First, if a sequence of ejection events occurs in a time span over which the asteroid is not able to relax to uniform rotation, the important quantity is the total accumulation of the angular momentum, or

(40) $$\Delta H_{T}=\overset{N}{\underset{i=1}{\sum }}\Delta H_{i}.$$

Then, if the events can be characterized as having a mean value $\bar{\Delta H}$ and a variance $\sigma_{\Delta H}^{2}$, then the expected value and variance of the total accumulated angular momentum change is ${\bar{\Delta H}}_{T}=N\bar{\Delta H}$, and its variance $\sigma_{\Delta H_{T}}^{2}=N\sigma_{\Delta H}^{2}$. Thus, even if the expected value of the total angular momentum has zero mean, we see that the variance of the values will steadily grow over time, making larger and larger deviations from the zero mean value more likely. Thus, in terms of the first‐order contributions, we see that even though the mean value of $\Delta H_{zT}$ contribution may be zero, its variance grows as $\sigma_{\Delta H_{zT}}∼\sqrt{N}\sigma_{\Delta H_{z}}$. The case is similar for the other angular momentum components. Then the modified spin state follows equations (15) and (16):

(41) $$\Omega_{l}∼\Omega +\frac{\Delta H_{zT}}{I_{zz}}+\frac{1}{2I_{zz}^{2}\Omega }\left[\frac{2I_{zz}-I_{xx}}{I_{xx}}\Delta H_{xT}^{2}+\frac{2I_{zz}-I_{yy}}{I_{yy}}\Delta H_{yT}^{2}\right]+\ldots ,$$

(42) $$I_{D}∼I_{zz}-\frac{1}{I_{zz}\Omega^{2}}\left[\frac{I_{zz}-I_{xx}}{I_{xx}}\Delta H_{xT}^{2}+\frac{I_{zz}-I_{yy}}{I_{yy}}\Delta H_{yT}^{2}\right]+\ldots .$$

As the angular momentum change accumulates and varies, these terms also vary. If the total change in angular momentum occurs over an active period of time, followed by a longer period of inactivity, the spin state of the system may relax. Then the ultimate change in spin rate would be

(43) $$\Delta \Omega ∼\frac{\Delta H_{zT}}{I_{zz}}+\frac{1}{2I_{zz}^{2}\Omega }\left[\Delta H_{xT}^{2}+\Delta H_{yT}^{2}\right]+\ldots ,$$

following equation (17).

If instead we assume that the asteroid dissipates its complex motion and settles back into uniform rotation between each ejection event, then the overall change in the total rotation rate for a single event is

(44) $$\Delta \Omega_{i}=\frac{\Delta H_{zi}}{I_{zz}}+\frac{1}{2I_{zz}^{2}\Omega }\left[\Delta H_{xi}^{2}+\Delta H_{yi}^{2}\right]+\ldots .$$

Now, we wish to compute the expected value of the sum $\Delta \Omega_{T}=\sum_{i=1}^{N}\Delta \Omega_{i}$. We can again assume that the mean of $\bar{\Delta H}=0$, but we notice (assuming a Gaussian distribution for the probability density function) that $\bar{\Delta H_{x}^{2}}=\sigma_{\Delta H_{x}}^{2}$, and thus, the expected value of the change in spin rate is

(45) $${\bar{\Delta \Omega }}_{T}=\frac{N}{2I_{zz}^{2}\Omega }\left[\sigma_{\Delta H_{x}}^{2}+\sigma_{\Delta H_{y}}^{2}\right].$$

Similarly, its variance will also grow over time as

(46) $$\sigma_{{\bar{\Delta \Omega }}_{T}}=\sqrt{N}\frac{\sigma_{\Delta H_{z}}}{I_{zz}}+\ldots ,$$

meaning that the change in spin rate can take on an increasing variation over time. Thus, the spin rate will inexorably grow, albeit potentially at a slow rate.

### 6.2 Uniform Loss

The added angular momentum is simply a function of surface location and body‐relative velocity and is independent of the spin state of the body. Whether or not a particle escapes is a strong function of the ejection speed and location, but the angular momentum removed from the main asteroid body is not. Thus, if we assume a uniform pattern of location and, more importantly, ejection direction about the asteroid, then we can note some specific results.

Let us assume the simplest model for ejection. The rate at which particles are ejected from the surface is proportional to the area of the surface, and the ejection of these particles will in general occur normal to the surface. Thus, suppose that each facet of area $A_{i}$ contributes an angular momentum according to a surface density Δσ and ejection speed v as

(47) $$\Delta H_{i}=-\Delta \sigma \;v\;A_{i}r_{i}\times {\hat{n}}_{i}.$$

Consider the projection of this onto a fixed direction, in general defined as $\hat{z}$. Then we find

(48) $$\Delta H_{zi}=-\Delta \sigma \;v\;A_{i}\hat{z}\cdot (r_{i}\times {\hat{n}}_{i})$$

(49) $$=-\Delta \sigma \;v\;\left(\hat{z}\times r_{i}\right)\cdot A_{i}{\hat{n}}_{i}.$$

In general, to obtain the entire contribution of the angular momentum along this direction, we sum this over the surface of the asteroid, to find

(50) $$\Delta H_{z}=-\Sigma_{i\in S}\Delta \sigma \;v\;\left(\hat{z}\times r_{i}\right)\cdot A_{i}{\hat{n}}_{i}.$$

Here the speed is fixed at a given value, as is the density. In the limit, this summation can be reduced to the surface integral

(51) $$\Delta H_{z}=-\Delta \sigma \;v\;\int_{S}\left(\hat{z}\times r\right)\cdot \;dS,$$

where dS is the differential surface element, oriented normal to the surface and with magnitude equal to the differential surface area.

For this situation, we can apply Stokes' theorem. The quantity $-\hat{z}\times r$ can be written as the curl of a vector function $F=\frac{1}{2}\left(x^{2}+y^{2}\right)\hat{z}$. Then, applying the theorem to the above integral, we can reduce the computation to the line integral over the boundaries between where ejection occurs and where it does not.

(52) $$\Delta H_{z}=\Delta \sigma \;v\;\int_{\Gamma }F\cdot d\Gamma$$

(53) $$=\frac{1}{2}\Delta \sigma \;v\;\int_{\Gamma }\left(x^{2}+y^{2}\right)\hat{z}\cdot d\Gamma .$$

Similar equations can be derived for the total angular momentum contribution to the other directions. Here the path Γ consists of all the surface contours that separate regions that escape and thus contribute their angular momentum to the body and regions that eventually fall back to the surface and need to be budgeted elsewhere. This equation is not necessarily the easiest way to compute the net contribution of the angular momentum. For the general contribution, it is easiest to sum up the contribution over each surface facet of a shape model, making the assumption that the net torque can be evaluated at the center of each facet.

In the limit, when the entire surface is ejecting material that immediately escapes, then the boundary of the region between ejection and nonejection vanishes, and thus, the integral goes to zero. Alternatively, one can construct this result using the faceted surface, assuming that each facet is considered on its own and that each facet ejects mass uniformly. Then, carrying out the surface integral over each facet, for every edge that is transited in a given direction, the neighboring edge will similarly be transited, nulling the contribution and yielding zero contribution to the angular momentum. The same conclusion can be reached for uniform ejections in the $\hat{x}$ and $\hat{y}$ directions. Thus, if the body is, on average, emitting particles uniformly from its surface and they all escape, the net effect on its rotation will be null.

### 6.3 Uniform Mass Migration

An important component are those particles that are ejected yet fall back to the surface eventually. In the following discussions, we ignore the effect of exogenous forces, although these should be considered in future work.

Let us assume that the particles are uniformly ejected from the asteroid surface and deposited onto the equator, which is seen as the most likely region for impact based on detailed numerical propagation of particles (McMahon et al., 2019). In the following, we just use a simple spherical moment of inertia to develop the main, qualitative effects of such a migration. The $I_{zz}$ moment of inertia of a surface shell of total mass ΔM is $2/3\Delta MR^{2}$. If this total mass is redeposited in a ring at the equator, the new contribution to the moment of inertia is $\Delta MR^{2}$. Thus, the overall change in moment of inertia is $1/3\;\Delta MR^{2}$. Then, the overall change in spin rate is computed from the balance $\Delta I_{zz}\Omega =-I_{zz}\Delta \Omega$. Using our simple spherical moment of inertia formula, we find that $\Delta \Omega /\Omega =-\Delta I_{zz}/I_{zz}=-5/6\Delta M/M$. So, given an estimate of the net rate of transfer of particle mass to the equatorial regions, we can find an estimate for a net change in spin rate, which generally slows the asteroid rotation rate.

To generate the same magnitude of the observed change in spin rate by mass migration (ignoring the opposite signs) requires the transfer of $\Delta M∼1\times 10^{-3}$ kg/s, or about a 1‐cm particle ejected per second. Compared with the mass redistribution rate of $10^{-7}$ kg/s documented by observation, this could account for ∼0.01% of the magnitude of the YORP effect. There is a real possibility that the mass redistribution rate could be larger than the mass loss rate, especially if low‐speed ejecta predominate in a steeper size distribution.

### 6.4 Uniform Ejection at a Given Speed

The above computations assume that every particle that is ejected escapes or reimpacts. However, for low enough speeds, this is not the case, and we must deal with both escape and mass redistribution. At every point of the surface, there is a speed above which the particle will be immediately ejected and below which it is likely to fall back onto the surface. One way to track this is to apply the above model but now explicitly track the ejection speed across the surface. Following the discussion above, we consider the effect of ejected particles uniformly across the surface at a given speed v.

If the particle escapes, we can add the magnitude $\Delta H_{z}=-m\hat{z}\cdot (r\times v)$. If the particle has less than escape speed, we assume that it migrates down toward the equator. To be conservative, we assume that all nonescaping ejecta migrates to the equator—which is certainly possible energetically on Bennu. This conserves angular momentum on Bennu but changes its spin rate. To track this, we compute the net change in spin rate for a given speed and location as $\Delta \Omega =-\Omega \;m\left(R_{H}^{2}-r_{H}^{2}\right)/I_{zz}^{B}$, where $r_{H}$ is the horizontal radius at the ejection point and $R_{H}$ is the equatorial radius to which the particle migrates. Here we use the average equatorial radius, equal to 270 m, for our computations.

**Figure 3:**
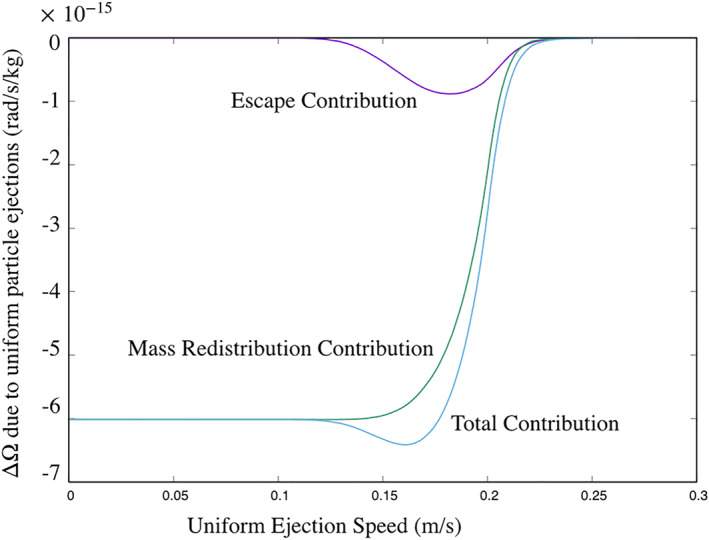
Change in spin rate due to uniform particle ejection at a given speed, accounting for escape and particle redistribution.

These effects are combined in the computation shown in Figure 3. Here it is assumed that the entire surface experiences a given ejection speed, with the mass lost proportional to the facet area. If the particle escapes, its contribution to the overall angular momentum is summed, to be evaluated later. If the particle reimpacts, the corresponding change in asteroid spin rate is summed. For low speeds below the escape limit across the entire body, the net change in spin rate is only due to redistribution. With our conservative model, this contribution remains constant until a more sizable number of particles begin to escape. As the ejection speed rises, particles facing the East are preferentially ejected, as the surface normals are tilted in their direction. This leads to an initial decrease in spin rate, which persists as ejection speeds rise. Also, as the number of escaping particles rises, the effect of redistribution becomes more muted. The maximum effect occurs at an ejection speed of 18 cm/s, at which point particles are ejecting from about 50% of the surface area. As the ejection speed rises, the amount of surface area with escaping particles increases, and, per the results above, the net change in spin rate starts to decrease until it reaches zero, when the entire surface loses its ejected particles. Figure 3 shows this computation.

The amount of mass needed to be ejected as a function of time to reach the magnitude of the YORP effect is about $8.5\times 10^{-3}$ kg/s, or about eight 1‐cm particles per second. This is well above the observed ejecta mass escape and redistribution rate of eight 1‐cm particles per day, making it a small contribution to the observed YORP spin acceleration.

### 6.5 Nonuniform Ejection

The effect of ejections become much more meaningful if they occur in a systematic direction on the surface. To bound the problem, we evaluate an extreme situation where the particles are launched due east or due west from the equator with a given speed greater than the local escape speed. If the ejections are not uniform, they will not average out over the body. A single ejected particle will generate a change in angular momentum of $\Delta H_{z}$ of −mRv if it launches to the East, and of +mRv if it launches to the West. Assuming this goes entirely into the spin, we can compute the fractional change in spin rate as $\Delta \Omega /\Omega ∼\pm \frac{mRv}{\Omega I_{zz}^{B}}∼\pm v\left(4.5\times 10^{-14}\to 4.5\times 10^{-11}\right)$. For simplicity, we can take an ejection speed of 1 m/s, within the range of velocities reported by Lauretta, Hergenrother, et al. (2019). Again, equating this to the YORP effect requires the ejection of one 0.5‐cm particle every 0.5 s, one 1‐cm particle every 4 s, or one 5‐cm particle every 7.5 min.

Comparing this to the documented ejection rate of 8‐cm‐sized particles per day, this implies that the maximum effect of nonuniform ejection can at most account for ∼0.04% of the measured YORP effect. If we instead allow for 10 m/s ejection speeds, this increases to 0.4%, more significant but still small. The rate of particle loss is highly uncertain, however.

We can also consider the effect of nonuniform mass ejection that induces off‐spin axis changes in angular momentum, which would then cause the system to relax, increasing the overall spin rate as in equation (44). This is a second‐order effect and thus is less efficient in changing spin rate than direct, nonuniform ejection. Equating the rate of YORP spin up over 1 s to the change in spin rate due to relaxation allows us to find the necessary rate of transverse angular momentum to account for YORP. This gives

(54) $$\Delta H_{T}∼H\;\sqrt{{\dot{\Omega }}_{Y}/\Omega \;\Delta t},$$

which over 1 s gives a $\Delta H_{T}∼10^{5}$ kg m ${}^{2}$/s. Assuming ejection of particles normal to the equator at 1 m/s, this would require 450 kg/s, much beyond anything reasonable.

**Figure 4:**
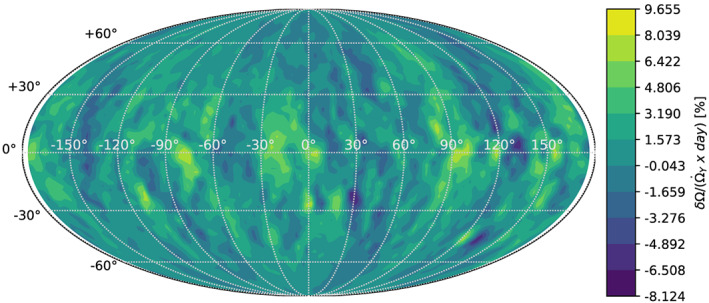
Angular velocity change due to a 10‐cm particle ejected at 1 m/s normal to surface, over the change caused by 1 day of YORP acceleration.

Larger particle events could have some significance for the changing spin rate according to their location on the surface. Figure 4 presents the change in Bennu's angular velocity due to a 10‐cm particle (1 kg) ejected at 1 m/s normal to the surface location, compared to the change caused by 1 day of the YORP effect. The figure shows a heatmap of all possible ejection locations on the surface, with maximum changes to the angular velocity equaling ∼10% of the daily YORP change. A preference for spin‐up can be seen on west facing slopes, and a preference for spin‐down can be seen on east facing slopes. If these contributions are summed over the entire body, accounting for the area within each region, the net contribution is zero, as expected given our previous discussion.

## 7 Discussion

### 7.1 Generality of the Particle YORP Model

In the current analysis the cause of these particle ejections has not been considered; however, the methodology shown here can be applied generally to a number of the different hypothesized processes that lead to particle ejection. Lauretta, Hergenrother, et al. (2019) propose several possible causes, including thermal fracturing, phyllosilicate dehydration, and meteoroid bombardment. The current model can be applied to each of these, albeit with some modification. The overall strength of the particle ejection effect may vary depending on the cause. We briefly discuss each of these effects in the following.

Thermal fracturing causes material to be spalled off of boulders and directly ejected across and away from the asteroid surface. This leads to the ejection of discrete mass particles, precisely as modeled herein. Thus, once the appropriate rates and speeds of mass ejection are combined with any systematic trends in surface orientation, the results of our computations can be directly applied, largely without modification.

Phyllosilicate dehydration is a chemical transformation process. The general model of this event would also lead to the ejection of gas from the surface, in addition to individual particles. Gas ejection is not explicitly modeled in this analysis, yet the model can account for it with a small modification. One can reasonably assume that the majority of ejected gas components would immediately be placed on an escape trajectory and thus would not have further interactions with the asteroid. In this case, it is possible to model the gas component of the release as a single particle of equivalent momentum being ejected from the surface, following the analysis in section 5.4. This would be added to the ejection of solid particles from the surface, for which the analysis already accounts. If this effect occurs uniformly over the surface (or uniformly in longitude across a band of latitudes), the additional contribution from gas ejection would in general average to zero in terms of its impact on the spin rate. If there are systematic surface orientations at which this process occurs, then there may be a net additional contribution to the body spin rate evolution.

In the case of meteoroid impacts, the ejected particles are from crater excavation, and the situation is more similar to that analyzed for the angular momentum drain effect (Dobrovolskis & Burns, 1984), and which is also specifically studied in Wiegert (2015) where it is found that this effect should only be important for bodies tens of meters in size. The analysis presented here can be used to analyze this situation but only captures the ejected component of the momentum transfer. To capture the effect of the impactor's momentum, one would need to compute the net momentum applied to the asteroid and sum it with the net ejected component. The ratio of the ejected momentum to the impact momentum can range from near 0 to a factor of several (Housen & Holsapple, 2012). Each impact would then add an additional angular momentum component of ΔH=mr×v, where r is the location where the impact occurs and v is the velocity of the impacting body. As analyzed in Dobrovolskis and Burns (1984), the averaged effect of these impacts by themselves will tend to zero, as each impact is as likely to add or subtract momentum from the body. The ejected particles would still have the most control over the net change in body angular momentum. If the ejecta speeds straddle the escape speed, then there can be a net contribution to the angular momentum. However, if they all escape, then we have the situation shown in Figure 3, where the net change averaged over the surface is zero.

Thus, we see that the analysis presented here can be generalized to describe the different physical theories for particle ejection. Any future improvement in our knowledge of the precise mechanism and the true flux of ejected particles could change some of the immediate conclusions and analyses presented here. For example, if the ejections are thermally driven, it may be reasonable to expect that western facing boulders would preferentially eject particles (due to them being warmer in general Golubov & Krugly, 2012), which would tend systematically to spin‐up the asteroid. Conversely, if the ejections are due to meteorite impacts, and if there is a sizable flux of ejected particles with speeds in the vicinity of the escape speed from Bennu, the particles ejected to the east will preferentially escape while the particles ejected to the west will not, which would tend to slow the asteroid spin rate. Due to the uncertainty of which mechanism is the cause of particle ejection, such refined computations and analyses of the effect cannot be made currently.

### 7.2 Geophysical Implications of Mass Redistribution

**Figure 5:**
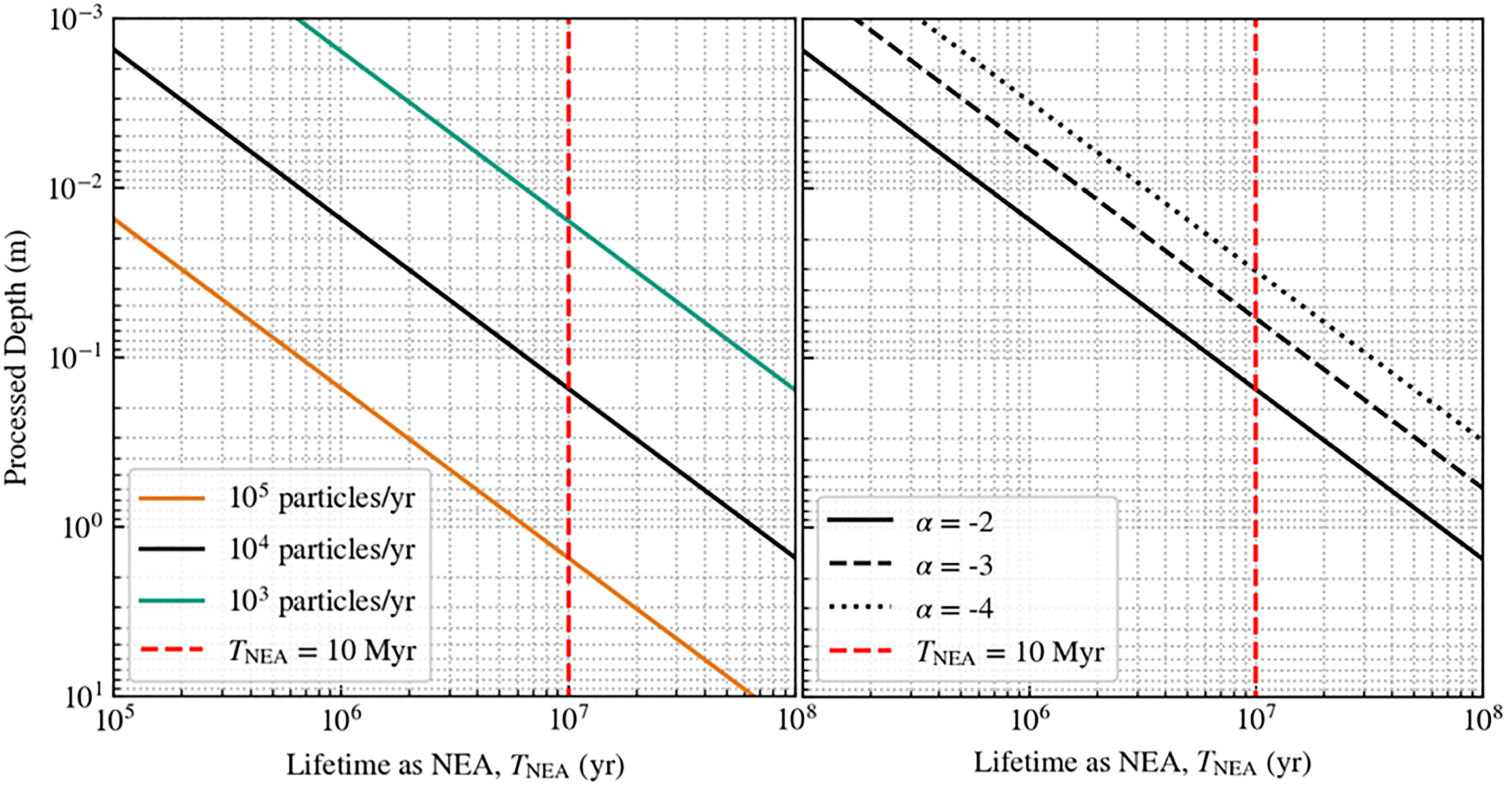
The integrated depth of Bennu's surface that is processed from particle ejection events. Left panel: for a power law cumulative particle size frequency distribution with exponent, α=−2, we show the processed depth for various total number of particles ejected per year. Assuming that these particle ejection events began when Bennu became a NEA, the top ∼2 m can be processed in 10 Myr (vertical red dashed line). Right panel: We evaluated the effect of varying the size distribution exponent, since this has yet to be well characterized for the observed ejected particles (Lauretta, Hergenrother, et al., 2019). We find that this has a notable effect on the processed depth, but variations are within 1 order of magnitude for the likely range evaluated.

One of the key predicted results from the existence of particle ejection is the redistribution of material over the surface. The general effect of this has been outlined above; thus, it is relevant to perform some additional modeling to ascertain the extent to which resurfacing may occur. There are two potential effects of this process on Bennu's geological properties through this ballistic redistribution of material: (1) surface homogenization of the top‐most regolith layer or (2) accumulation of fine particles in preferred regions of reimpact. To evaluate whether this is a macroscopic effect, we integrate the total volume of particles ejected from the surface over Bennu's probable lifetime in near‐Earth space using the model in section 2.2, $T_{NEA}\in [1,100]$ Myr (Delbo & Michel, 2011). Figure 5 shows the accumulated effect of particle ejections on the surface of Bennu for different assumed ejection rates. We find that for α=−2, and a particle ejection rate between $10^{4}$ and $10^{5}$ particles per year processes the top ∼0.2–2 m of the surface in 10 Myr.

The accumulated effect of this processing on Bennu's macroscopic structure depends on the dynamical fate of the ejected particles and competing geologic effects, such as impact cratering from micrometeorites or mass flow (Walsh et al., 2019). Ignoring these competing effects for the moment and assuming that most of this population reaccumulates on Bennu, a random reimpacting of the surface would suggest that the upper tens of centimeters of Bennu's surface would be homogenized in 10 Myr. However, if there is an equatorial preference for particle reaccumulation, as described by McMahon et al. (2019), then we would expect that tens of centimeters of material from the midlatitudes and poles would be ballistically redistributed to the equator (which occupies roughly half of Bennu's total surface area), suggesting an accumulation of small particles ( <5 cm) in these regions. There is little direct visual evidence for such a latitudinal size sorting of particles on Bennu (Walsh et al., 2019); thus, other geologic processes such as radial size sorting or impact excavation may have dominated over this process and depleted the equator of this fine layer of particles. However, the existence and persistence of this transfer process may explain the slope dichotomy seen in the equatorial region, within Bennu's rotational Roche lobe, as explored by Scheeres et al. (2019). The predicted redistribution of material ∼1 m in depth over 10 Myr represents a relative mass shift ΔM/M∼3ΔR/R∼0.012. If concentrated toward the equator, it would lead to a relative change in spin rate of ΔΩ/Ω∼−0.01. Given that the YORP timescale is estimated to be 1.5 Myr, we see that this effect is not a dominant process at the currently estimated levels of particle ejection.

## 8 Conclusions

The phenomenon of particle ejection‐driven changes in asteroid spin and orbit drift rates is studied in the context of the asteroid Bennu, which has newly been determined to be an “active asteroid.” The effect of particle ejection on orbital drift rate as compared to the Yarkovsky effect is analyzed and confirmed to be negligible. The same is not necessarily true for the spin rate of the asteroid, where certain systematic ejection patterns could lead to a relatively large effect on Bennu's spin rate evolution. Even so, most of the ejection effects will tend to decrease the asteroid total angular momentum and thus decrease its spin rate. Thus, in the context of Bennu, these findings imply that the net YORP effect may be larger than previously documented. However, given the measured ejection and redistribution rates, the effect on asteroid spin appears to be minimal, less than 1%. Our study also finds that mass redistribution due to low‐speed ejection events can be an important influence on the spin rate and can compete with particle ejection and escape. The general analysis developed herein can be applied to several of the different models for particle ejection. The results of this study can be applied to other bodies that may experience similar mass loss events, even if with substantially different ejection rates. While the overall effect of particle ejections are found to be modest for Bennu given the measured rates, their analysis further quantifies the nongravitational effects, which can modify a small asteroid's spin rate.
